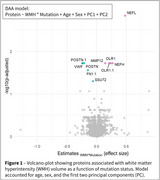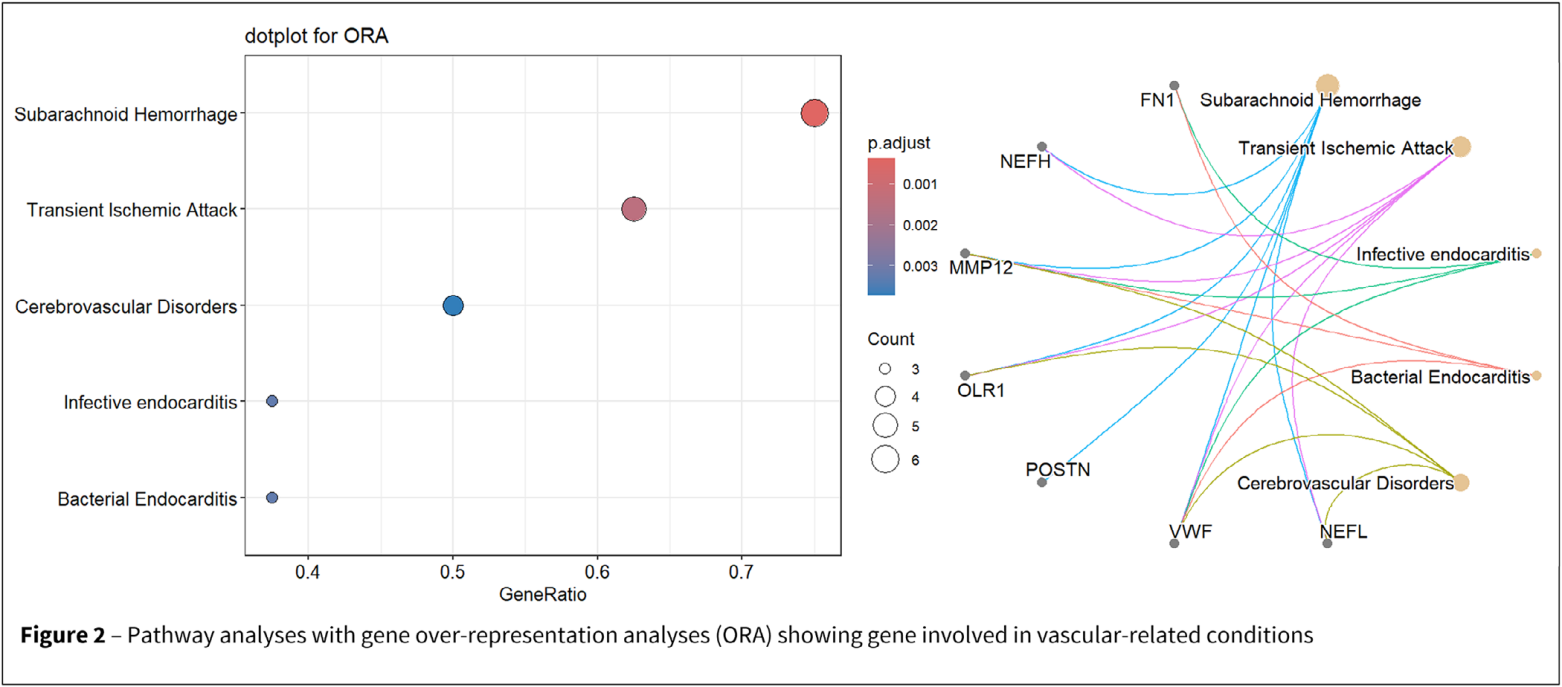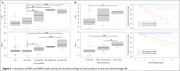# Elevated CSF levels of matrix metalloproteinase‐12 as a potential marker for microhemorrhage risk in autosomal dominant Alzheimer disease

**DOI:** 10.1002/alz70862_109993

**Published:** 2025-12-23

**Authors:** Nelly Joseph‐Mathurin, Katherine Gong, Gengsheng Chen, Parinaz Massoumzadeh, Jeremy F. Strain, Laura Ibanez, Brian A. Gordon, Jorge J. Llibre‐Guerra, Jason J. Hassenstab, Richard J. Perrin, Chengjie Xiong, Randall J. Bateman, Eric McDade, Tammie L.S. Benzinger, Carlos Cruchaga

**Affiliations:** ^1^ Washington University School of Medicine, St. Louis, MO USA; ^2^ Washington University School of Medicine in St. Louis, St. Louis, MO USA

## Abstract

**Background:**

With the advent of disease‐modifying treatment for Alzheimer disease (AD), identifying biomarkers for predicting risk for amyloid‐related imaging abnormalities (ARIA), hemorrhagic or edema types, is of increased interest. ARIA are thought to be related to disruption of the blood‐brain barrier as fibrillary amyloid is cleared from the brain. Molecular and cellular processes related to these events may inform future trials. We investigated proteomics related to abnormal neurovascular imaging phenotypes such as white matter hyperintensities (WMH) in autosomal dominant AD (ADAD), a relatively young population at risk for ARIA.

**Methods:**

Participants from the Dominantly Inherited Alzheimer Network observational study (n_Carriers_=290 and n_Non‐Carriers_=183) were assessed for WMH and microhemorrhages using T2‐FLAIR and T2*GRE MRI, and for CSF proteomics using the 7k Somalogic^®^ platform. A subset (n_Carriers_=92 and n_Non‐Carriers_=51) was evaluated for microhemorrhage incidence. WMH volumes were segmented with Triplanar U‐Net ensemble network. Microhemorrhage count and incidence were classified as none, mild, moderate, or severe, based on current FDA recommendations. We performed differential abundance analyses to investigate proteins associated with WMH as a function of mutation status, accounting for age, APOE‐e4 status, and sex, and significant proteins were further evaluated in pathway analyses and for associations with microhemorrhages.

**Results:**

Eight proteins were differently expressed in carriers with larger WMH volumes (Figure 1). The genes of seven proteins (e.g., neurofilament light‐chain (NEFL), neurofilament heavy‐chain (NEFH), matrix metalloproteinase 12 (MMP12), fibronectin‐1 (FN1), periostin (POSTN)) were overly represented in vascular‐related disorders such as subarachnoid hemorrhages, transient ischemic attack, or cerebrovascular diseases (Figure 2). CSF levels of NEFL, NEFH, MMP12, fibronectin1, and periostin differed as a function of CMH severity. Especially, NEFL and MMP12 were higher in carriers with severe CMH compared to those with none or mild CMH (Figure 3A). MMP12 levels were particularly high in participants having severe increase in microhemorrhages (Figure 3B). Carriers with high levels of MMP12 may more likely develop new microhemorrhages.

**Conclusions:**

Our findings confirm the contribution of neurofilament light chain in disease processes and suggest a role for matrix metalloproteinase 12 in the development of microhemorrhages and especially severe case in ADAD.

**Funding**: K01AG080123, RF1‐AG044546, UF1AG032438